# The effect of position on the precision of dual-energy X-ray absorptiometry and correlation with body condition score in dogs and cats

**DOI:** 10.1017/jns.2017.16

**Published:** 2017-05-15

**Authors:** Charlotte R. Bjørnvad, Mie E. Nielsen, Susanna E. M. Hansen, Dorte H. Nielsen

**Affiliations:** Department of Veterinary Clinical and Animal Sciences, Faculty of Health and Medical Sciences, University of Copenhagen, Dyrlaegevej 16, 1870 Frederiksberg C, Denmark

**Keywords:** Canine nutrition, Feline nutrition, Body composition, Fat percentage, Lean body mass, BCS, body condition scoring, BF, body fat, BF%, body fat percentage, BMD, bone mineral density, BW, body weight, DEXA, dual-energy X-ray absorptiometry, TBM, total body mass

## Abstract

Dual-energy X-ray absorptiometry (DEXA) has been used to assess body composition in dogs and cats in several studies, but studies are difficult to compare for several reasons. The aim of the present study was to evaluate whether positioning of dogs or cats in either dorsal or ventral recumbency during DEXA scanning influences results. Dogs and cats that were brought to the University Hospital for Companion Animals for euthanasia during the period 15 September–6 November 2015 were consecutively recruited if owners signed a written consent. Following euthanasia and before rigor mortis, the animals were body condition scored (BCS, nine-point scale) and DEXA scanned. DEXA measurements of total body mass (TBM), bone mineral content (BMC), bone mineral density (BMD), lean soft tissue mass (LSTM) and body fat (BF) were performed five times in ventral and two times in dorsal recumbency on each animal. Differences between positioning were analysed using Student's *t* test or Wilcoxon's test depending on normality of the data. A total of thirteen dogs and seven cats of different breeds, size, sexes and age were included. The CV for DEXA parameters in ventral or dorsal recumbency were, for dogs, TBM ≤ 0·1 %, BMC ≤ 1·63 %, BMD ≤ 1·29 %, LSTM ≤ 0·89 % and BF ≤ 1·52 %; and, for cats, TBM ≤ 0·08 %, BMC ≤ 0·61 %, BMD ≤ 0·49 %, LSTM ≤ 0·45 % and BF ≤ 0·88 %. In both positions, a good correlation was found for dogs (*r* 0·84–0·85; *P* < 0·0003) and cats (*r* 0·89–0·90; *P* < 0·0081) between the nine-point BCS system and BF percentage measured by DEXA. Ventral and dorsal recumbency provides comparable results, except that BMD measures were higher in dorsal recumbency (*P* < 0·0004).

Obesity is the most common nutritional disorder encountered in companion animal veterinary practice^(^^1^^)^. To assess body composition and identify dogs and cats at risk of obesity as well as estimating the degree of obesity, reliable and accurate methods are needed. In obesity research where results often are related to degree of obesity it is essential that the assessment of body composition, on which calculations and conclusions are based, is as precise and accurate as possible.

The ‘gold standard’ for body composition analysis is chemical analysis of a carcass, but as this is seldom an acceptable methodology in clinical research, dual-energy X-ray absorptiometry (DEXA) is often considered one of the best alternatives^(^^2^^,^^3^^)^. DEXA has been used in several obesity studies on dogs and cats and it generally performs well with low variance for most measurements^(^^2^^–^^10^^)^. However, these studies are difficult to compare, both because different machines and software have been used, but maybe also due to differences in the investigators’ preference for positioning of the animal during scanning^(^^6^^,^^7^^)^. In clinical situations where DEXA is not available, body condition scoring (BCS) methods such as the nine-point BCS system are often used for assessment of body composition in dogs and cats^(^^4^^,^^5^^,^^10^^)^. They are generally less accurate, categories overlap and the measured body fat (BF) percentage (BF%) relating to each BCS differs between studies and populations investigated^(^^3^^–^^9^^)^. However, they seem to be useful in identifying overweight in pets and, further, for planning of weight-loss programmes for dogs and cats^(^^11^^)^. The degree of energy restriction is often calculated based on estimated ideal body weight (BW), which is assessed by relating BCS to DEXA-estimated degree of overweight^(^^11^^)^. From BCS4 to BCS9 on a nine-point scale, each incremental increase in BCS has in many studies been shown to equal an increment of 10–15 % in DEXA-measured BF%^(^^4^^,^^5^^,^^7^^,^^9^^,^^10^^)^. The aims of the present study were to: (1) evaluate the precision of the DEXA scanner at the University Hospital for Companion Animals, Copenhagen, Denmark; (2) evaluate the effect of positioning in either dorsal or ventral recumbency; and finally (3) to evaluate correlation between DEXA-measured BF% and a nine-point BCS system.

## Experimental methods

### Animals

Client-owned dogs and cats that were brought to the University Hospital for Companion Animals for euthanasia for other reasons during the period 15 September–6 November 2015 were consecutively recruited. Animals were only included if owners signed an informed consent accepting that their animal could be used for research and teaching purposes following euthanasia. The study was approved by the local ethical and administrative committee at the department before initiation.

### Dual-energy X-ray absorptiometry scanning

Following euthanasia and before rigor mortis, the dogs and cats were weighed on a scale (dogs: Soehnle 7742, Soehnle-Waagen; cats: Kruuse MS-20, Kruuse), body condition scored^(^^4^^,^^5^^)^ and DEXA scanned (Lunar Prodigy, GE Healthcare) using a small animal program in the ENCORE™ 2011 software (GE Healthcare, version 13.60). DEXA measurements of total body mass (TBM), bone mineral content, bone mineral density (BMD), lean soft tissue mass and BF were performed. Anticipating that rigor mortis would develop, we estimated that we would only have time to do seven scans and because ventral recumbency had not been evaluated previously we decided to perform scans five times in ventral and two times in dorsal recumbency on each animal. The animals were not repositioned between scans.

### Statistical analyses

For statistical analyses (GraphPad Prism, version 7; GraphPad Software Inc.), precision was evaluated based on CV on repeated measures. Differences between measurements obtained in ventral and dorsal recumbency were analysed using Student's *t* test or Wilcoxon's test depending on normality of the data and Pearson's correlation test was used to evaluate correlation between BCS and DEXA-estimated BF% for each position. Based on BF% measurements, which usually show the worst precision between measurements, power calculation showed that inclusion of five cats and five dogs would give a power of 0·99 with *α* = 0·05. Results are presented as median (range) and were evaluated as significantly different if *P* < 0·05.

## Results

A total of thirteen dogs were included, representing different breeds: three mixed breed and one of Shiba inu, Rottweiler, Shi tzu, golden retriever, miniature poodle, dachshund, Lhasa apso, French bulldog, Italian greyhound and Belgian shepherd. Of these, six were entire females, four entire males and three castrated males. The median age was 5·5 years (range 9 months–13 years), median BW was 15·2 (range 5·3–44·9) kg, median BCS was 6/9 (range 2–9), median BF% was 39·5 (range 9·6–63·4) %, median fat mass was 4·0 (range 1·0–15·8) kg, median lean tissue mass was 6·1 (range 2·5–28·8) kg and median BMD was 0·52 (range 0·36–0·75) g/cm^3^.

A total of seven cats were included: six domestic shorthair and one domestic longhair of which four were intact female, one spayed female and two neutered males. The median age was 10·6 (range 1·1–18) years, median BW was 4·6 (range 3·4–6·0) kg, median BCS was 5/9 (range 3–8), median BF% was 31·2 (range 10·8–73·4) %, median fat mass was 1·41 (range 0·37–3·03) kg, median lean tissue mass was 2·67 (range 1·10–3·96) kg and median BMD was 0·34 (range 0·32–0·39) g/cm^3^. There was generally a low variance between measurements in both ventral and dorsal recumbency (<2 %; Table 1). Ventral and dorsal recumbency provided comparable results, except for BMD measurements that were significantly higher in dorsal recumbency (*P* < 0·0004) while there was still a strong correlation (*r* 0·97; *P* < 0·0001). A strong association was found between BW measured on the scale with TBM, which was calculated based from the sum of bone mineral content, lean soft tissue mass and BF mass (*r* 1·00; *P* < 0·00001); however, TBM systematically underestimated measured BW by approximately 7 %.

**Table 1.** Variance of variables measured by dual-energy X-ray absorptiometry in ventral and dorsal recumbency, respectively, on dogs (*n* 13) and cats (*n* 7)* (Coefficients of variation and ranges)

	CV_ventral_ (%)	Range (%)	CV_dorsal_ (%)	Range (%)
Dogs				
TBM	0·10	0·05–0·27	0·09	0·01–0·19
LSTM	0·89	0·33–3·53	0·55	0·03–2·29
BF mass	1·52	0·33–4·20	0·79	0·03–2·02
BF%	1·56	0·55–4·12	0·80	0·00–2·16
BMC	1·63	1·01–6·60	0·95	0·00–2·80
BMD	1·29	0·45–2·70	1·21	0·25–6·77
Cats				
TBM	0·08	0·04–0·10	0·07	0·01–0·18
LSTM	0·45	0·26–0·85	0·14	0·07–0·26
BF mass	0·88	0·32–2·04	0·29	0·00–0·61
BF%	0·94	0·34–2·34	0·23	0·00–0·59
BMC	0·55	0·31–0·91	0·61	0·00–1·27
BMD	0·46	0·30–0·87	0·49	0·00–1·49

TBM, total body mass; LSTM, lean soft tissue mass; BF, body fat; BF%, body fat percentage; BMC, bone mineral content; BMD, bone mineral density.

* CV values of ventral recumbency are based on five consecutive scans and CV values of dorsal recumbency are based on two scans.

**Fig. 1.**
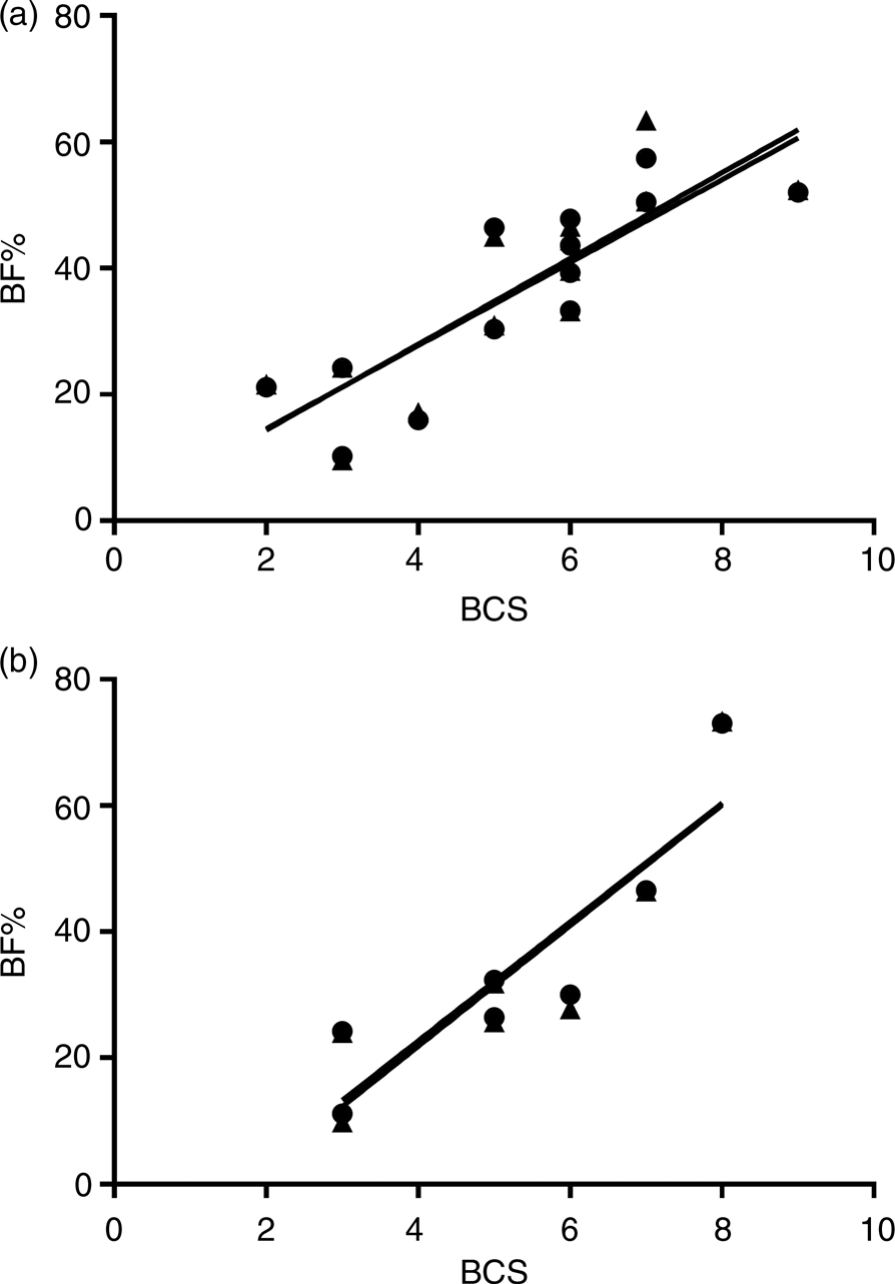
Correlation between body condition scoring (BCS; nine-point scale) and dual-energy X-ray absorptiometry-measured body fat percentage (BF%) measured in ventral (●) and dorsal (▲) recumbency on newly euthanised (a) dogs (*n* 13) and (b) cats (*n* 7).

In both positions, a good correlation was found for dogs (*r* 0·84 (dorsal)–0·85 (ventral); *P* < 0·0003) and cats (*r* 0·89 (dorsal)–0·90 (ventral); *P* < 0·0081) between the nine-point BCS system and BF% measured by DEXA, but the categories overlapped (Fig. 1). The mean BF% for BCS5 was 38 (sd 11) % in dogs and 29 (sd 4) % in cats and each BCS increment equalled an increase of 6·6 BF% in dogs and of 9·5 BF% in cats.

## Discussion

The present study showed that the DEXA machine (Lunar Prodigy; GE Healthcare) tested offers a precise method for evaluating body composition in dogs and cats of different size, age and breed. Ventral and dorsal recumbency provided comparable results, except for BMD. All CV values were generally low, but there seemed to be a tendency for even lower CV values when the animals where positioned in dorsal recumbency for many parameters. Dorsal recumbency may pose a problem during anaesthesia if the anaesthesia is prolonged or the patient is respiratory compromised. With the evolution of DEXA machines, a scan of a 30 kg dog takes about 10–20 min; it could therefore be considered if the lower CV value merits a standard placement in dorsal recumbency or if ventral recumbency is preferable because it only causes a limited increase in CV in this position.

Apart from precision, evaluating accuracy is another part of a proper method validation, which would require that we compared the results with a chemical analysis of the measured animals. This was unfortunately not possible because the animals were already reserved for other teaching purposes. However, accuracy has previously been evaluated for the pencil-beam Hologic QDR 1000/W DEXA machine in dogs and cats^(^^2^^)^. In that study the investigator found it to provide an accurate estimate of body composition in subjects weighing between 1·8 and 22·1 kg, but that individual discrepancies could be large and seemed to relate to hydration status. The CV values reported in our study were generally lower than reported previously for the Hologic QDR 1000/W densitometer^(^^9^^)^. However, the Lunar Prodigy scanner seems to have a better precision than the older Hologic scanner and has in a previous review on human subjects been reported to perform with CV <2 % for different tissues^(^^12^^)^. Further, comparing the DEXA-measured TBM with scale-measured BW serves to evaluate the overall accuracy of the DEXA method; we found a very good correlation between methods despite a slight systematic underestimation using DEXA compared with scale measurements. This underestimation has been reported previously and seems not to relate to just one body compartment^(^^2^^,^^13^^)^.

The correlation between DEXA and BCS has previously been reported to be between 0·69 and 0·92^(^^3^^,^^7^^,^^10^^)^. In one cat colony an ideal body composition (BCS = 3/5) equalled a BF% of 11·7 (sd 4·5) % in both male and female cats^(^^10^^)^, while in another cat colony a BCS of 5/9 equalled 21·8 (sd 1·7) % BF in male cats^(^^5^^)^ and in client-owned indoor confined cats a BCS of 5/9 was reported to equal about 30·1 (sd 4·1) % BF in male and 31·6 (sd 4·6) % BF in female cats^(^^7^^)^. The present study seems to agree that client-owned cats may have a relatively high BF% for a BCS 5/9. In dogs, an ideal body composition (BCS 5/9) corresponded to 11 (sd 2) % BF in twenty-three client-owned dogs^(^^3^^)^ while another study showed great variation depending on dog breed^(^^8^^)^ and in the present study we found a relatively high BF% relating to BCS 5/9. BCS should be used with caution in obesity research as well as for estimating BF%, as the extrapolated BF% seems to vary between populations.

Previously, one study has investigated positioning in dorsal and lateral recumbency during DEXA (Lunar Prodigy Advanced) in dogs and cats^(^^13^^)^. In that study, results were precise in both recumbencies but there were significant differences in the absolute measurements of fat and lean tissue mass between positionings, with scanning in dorsal recumbency reading more fat and less lean tissue relative to lateral recumbency. Because the DEXA methodology is only able to distinguish between two types of tissue at a time, bone *v.* soft tissue (lean and fat tissue together), or fat *v.* lean tissue when no bone is present, it is likely that ventral and dorsal recumbency give more similar results compared with dorsal and lateral recumbency.

Because we wished to perform the scans before rigor mortis developed and because ventral recumbency had not been evaluated before, we decided to only perform two scans in dorsal recumbency. This may have resulted in better CV values for dorsal recumbency compared with ventral, and the difference between the two may be even smaller. Another parameter that can influence DEXA results is age, as it may be expected that young animals have a larger muscle mass than older. We included animals of different ages in this study, with a higher proportion of older animals. However, this would not affect the precision of the individual measurements but could affect the correlation with the BCS system.

In conclusion, the Lunar Prodigy DEXA machine offers a precise technique for determining body composition in dogs and cats. The results from ventral and dorsal recumbency are comparable, although with a slightly higher precision and significantly higher BMD values in dorsal recumbency. The nine-point BCS system correlated well with DEXA measurements in both recumbencies but with overlapping results between categories.
